# Author Correction: Uncovering active precursors in colloidal quantum dot synthesis

**DOI:** 10.1038/s41467-018-02946-1

**Published:** 2018-02-09

**Authors:** Leah C. Frenette, Todd D. Krauss

**Affiliations:** 1 Department of Chemistry, University of Rochester, Rochester, NY 14627-0216 USA; 2 Institute of Optics, University of Rochester, Rochester, NY 14627-0216 USA

Correction to: *Nature Communications* 10.1038/s41467-017-01936-z, published online 12 December 2017

In the original version of this Article, Fig. 3 contained several errors in the chemical structures. The correct version is:

 **Fig. 1**

which replaces the previous incorrect version :

 **Fig. 2**

This has now been corrected in both the PDF and HTML versions of the Article.

