# Supplementary material for: Uncovering active precursors in colloidal quantum dot synthesis
Source: Nat Commun. 2017 Dec 12;8:2082. doi: 10.1038/s41467-017-01936-z (PMC5727186; doi:10.1038/s41467-017-01936-z)
Supplement: Supplementary file 1 — Supplementary Information [file 41467_2017_1936_MOESM1_ESM.pdf]

Supplementary Methods

Materials All compounds were used as received unless otherwise noted. Cadmium oxide (99.99%; CdO), oleic acid (90% tech. grade; OA), octanoic acid (99%), tetradecane (99%), selenium shot (99.999%), triphenylphosphine (99%; TPP), tri-*n*-butylphosphine (97%; TnBP), carbon disulfide (99.99%, CS_2), zinc selenide (99.99%, ZnSe) were purchased from Sigma-Aldrich. Toluene (99.9%), Hydrochloric Acid (ACS Grade) and acetic anhydride (99.6%) were purchased from Fischer Scientific. 1-^{13}C-Octanoic acid (1-13C, 99%), ^{13}C-Carbon dioxide (13C, 99%; $^{13}CO_2$), methylene chloride-*d2* (CD_2Cl_2), and toluene-*d8* were purchased from Cambridge Isotope Laboratories.

General Methods Chalcogens, toluene, and tetradecane were degassed and stored in a glovebox prior to use. All phosphines were also stored in a glovebox prior to use. Synthesized cadmium (Cd) precursors were degassed and kept under nitrogen before use in QD syntheses. All reactions were conducted under a nitrogen atmosphere. Reactions occurred in three-neck round bottom flasks, equipped with water-cooled condensers, under positive nitrogen pressure unless otherwise indicated.

Cadmium oleate synthesis Cadmium oleate stock solution (0.2 M) was synthesized according to previous reports: cadmium oxide (25 mmol, 0.318 g), oleic acid (100 mmol, 3.45 mL), and tetradecane, were combined in air. The solution was heated to 220 °C and allowed to react under nitrogen until the solution turned clear and colourless, approximately one hour. At room temperature the product was a white, waxy solid that needs to be heated above 60 °C before use.

Synthesis of selenium precursor Tri-*n*-butylphosphine selenide was prepared by mixing Selenium pellets with Tri-*n*-butylphosphine (99%) in toluene under a nitrogen atmosphere to make a 1M solution, heating at 60°C until homogeneous.

Tertiary phosphine selenide decomposition Tri-*n*-butylphosphine selenide (2 mmol) was injected into a flask of octanoic acid (4 mmol) in tetradecane (8 mL) at 250°C under a nitrogen atmosphere for 15 minutes. The resulting products in solution were analysed by ^{1}H, ^{31}P, and ^{77}Se NMR at different time points and carboxylic acid concentrations. A cannula transfer apparatus was assembled as in Figure 2b so that the gases from the TnBPSe decomposition flask were bubbled through the solution in the second flask. The gaseous products were collected in carbon disulphide (12 mL) at 0°C (ice bath) in the second flask. The solution reacted for 15 minutes, the amount of time necessary to make CdSe QDs in the same apparatus. The solution in CS_2 was then prepared in CD_2Cl_2 in a NMR tube with a STEM coaxial insert and a Triphenylphosphine selenide (TPPSe) external standard. A control experiment was conducted in parallel to allow for comparison of spectra; a CS_2 solution of hydrogen selenide was generated by reaction of zinc selenide with a dropwise addition hydrochloric acid in the first flask, as reported by Schneider and Wieghardt.[1] Additionally, the second flask was replaced with Cd oleate in tetradecane and resulted in the formation of CdSe nanocrystals.

Synthesis of cadmium octanoate ^{13}C labelled Cadmium octanoate was prepared by heating 1-^{13}C-Octanoic acid (4 mmol) and cadmium oxide (2 mmol) in tetradecane by heating to 210°C for 30 minutes in an schlenk flask. The resulting clear colourless liquid was degassed and the flask was sealed. The side arm of the flask was sealed with a septum and purged with nitrogen. The sealed flask was then heated to 280°C for 15 minutes, analogous to the conditions of a QD synthesis. The headspace of the reaction was analysed using GCMS. The purged space in the

arm of the flask was analysed to ascertain a background concentration of gasses. The Teflon pin of the flask was then opened allowing the headspace of the reaction to mix with the atmosphere in the arm of the flask. A second sample of gas was taken through the septum and the relative concentrations of gasses before and after mixing were determined. A calibration curve was created by varying the amounts of $^{13}CO_2$ added to a schlenk flask filled with an equivalent volume of tetradecane as in the experimental unknowns with an argon internal standard that was added equally across all samples. The ratio of counts versus the internal standard was calculated by dividing the counts of $^{13}CO_2$ (m/z=45) by those of Ar (m/z = 40). The average for each concentration was taken over five replicates.

NMR characterization Proton (^{1}H), carbon (^{13}C), phosphorus (^{31}P), and Selenium (^{77}Se) Nuclear magnetic resonance (NMR) spectra were recorded at ambient temperature on an Avance 500 (500 MHz) spectrometer (500.1 mHz for ^{1}H; 202.5 MHz for ^{31}P; 95.4 MHz for ^{77}Se; 125.8 MHz for ^{13}C). Chemical shift (δ) is recorded in ppm and coupling constants (*J*) are reported in Hertz (Hz).

GCMS characterization. Headspace samples were measured on a Shimadzu GCMS – QP2010. The instrument was operated with an injection temperature of 250°C, a column temperature ramping from 40-240°C, an ion source temperature of 225°C, and a column flow rate of 17 psi helium carrier gas at 1.05 mL/min

Supplementary Discussion

Thermal Decomposition of Tri-*n*-butylphosphine selenide

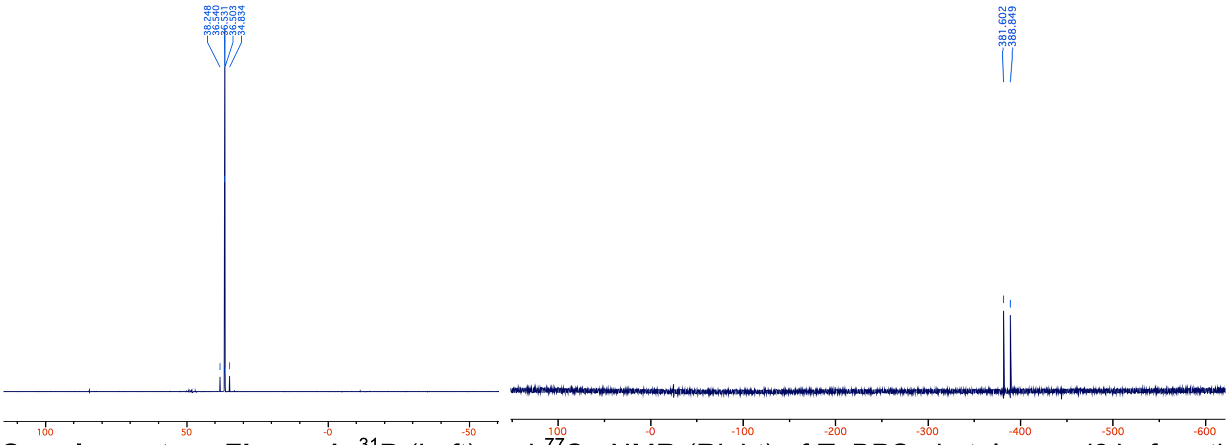

Supplementary Figure 1. ^{31}P (Left) and ^{77}Se NMR (Right) of TnBPSe in toluene-*d8* before the decomposition reaction. Chemical shifts are reported in Supplementary Table 1.

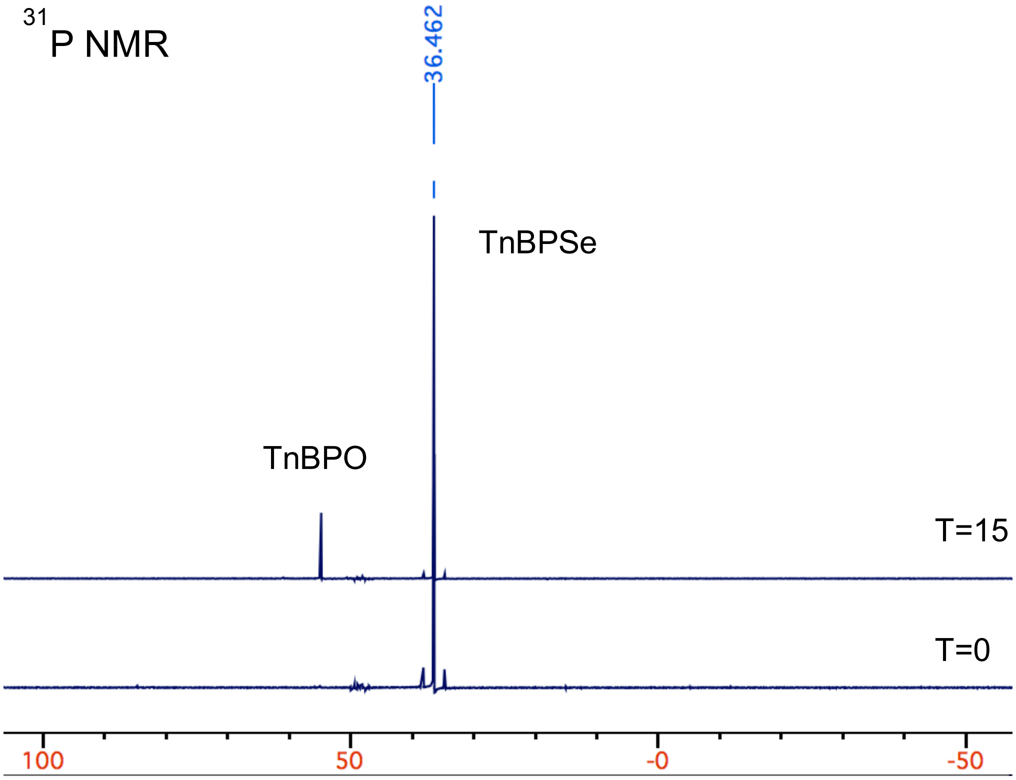

Supplementary Figure 2. ^{31}P NMR of TnBPSe in toluene-*d8* before and after 15 minutes of reaction at 250˚C, remaining in flask 1 of the cannula transfer apparatus. Chemical shifts are reported in Supplementary Table 1

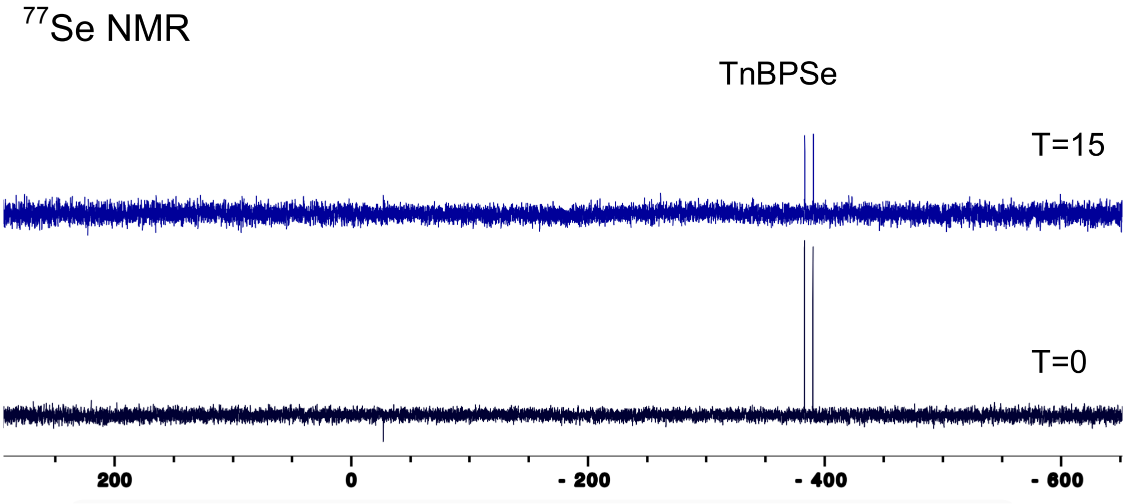

Supplementary Figure 3. ^{77}Se NMR of TnBPSe in toluene-*d8* before and after 15 minutes of reaction at 250˚C, remaining in flask 1 of the cannula transfer apparatus. Chemical shifts are reported in Supplementary Table 1.

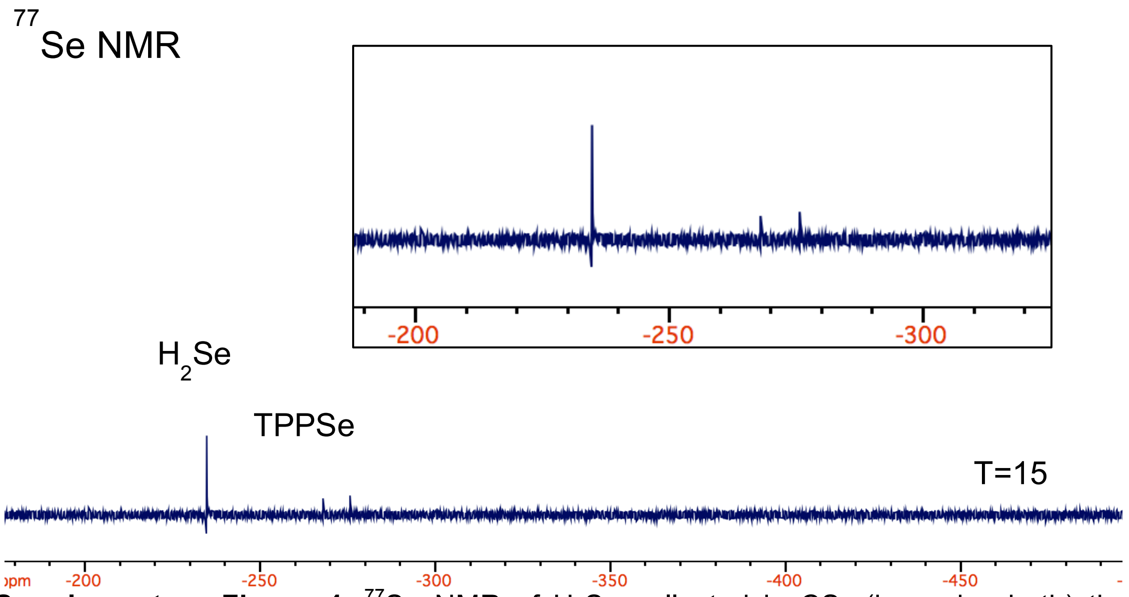

Supplementary Figure 4. ^{77}Se NMR of H_2Se collected in CS_2 (in an ice bath) through the cannula transfer experiment after 15 minutes of reaction at 250˚C and TPPSe in CD_2Cl_2, used an an external calibration standard. Inset: The region of interest on a larger scale. Chemical shifts are reported in Table S1.

An external calibration standard (TPPSe in CD_2Cl_2) was employed using a coaxial insert NMR tube. This provided a standard to compare relative intensities and relate concentrations without the possibility of the standard reacting with the species being studied. The external tube holds 490 μL of sample while the internal tube holding the reference holds 100 μL. The low concentrations resulted in low signal intensities coupled with the weak signal from ^{77}Se NMR, causing the ^{77}Se NMR experiments to be run for over 8 hours (overnight). The integrated peak intensities were compared to determine what percentage of TnBPSe was converted to H_2Se by calculating how much H_2Se was formed. The concentration of H_2Se was back calculated from the known concentration of the TPPSe external standard.

Supplementary Table 1. Chemical shifts of compounds by ^{31}P and ^{77}Se NMR

Compound	Experimental Shift (ppm)		Literature Shift (ppm)[2]
	{^{31}P}	{^{77}Se}	{^{77}Se}
TnBPSe	36.5	-384.9<br>d, *J*= 732 Hz	-384.6
TnBPO	53.7	-	-
TPPSe	35.2	-273.8<br>d, *J*= 707 Hz	-268
H_2Se	-	-231.1	-225.5
H_2Se Control	-	-228.5	-225.5

J(^{31}P, ^{77}Se) Coupling parameters are consistent with those seen by Alvarado *et al.* and consistent with what is expected from tertiary phosphine selenide compounds.[3]

Supplementary Note 1

The quantitative NMR spectroscopy[4] was calculated by relative quantitation of two compounds for determining the relative concentration of TnBPSe and TnBPO (Supplementary Equation 1) and absolute quantification for determining the amount of H_2Se generated from the known concentration of TPPSe external standard (Supplementary Equation 2). Because two different computational methods and spectroscopic methods were used to calculate the percent conversion of TnBPSe and H_2Se, there is error associated with each; the two calculations are not self-consistent. However, the average values are within 10% of one another (30% TnBPO and 36% H_2Se), which is acceptable given these experimental constraints.

Supplementary Equation 1

$$\frac{M_x}{M_y} = \frac{I_x}{I_y} \times \frac{N_y}{N_x}$$

Supplementary Equation 2

$$P_x = \frac{I_x}{I_{\mathrm{std}}} \times \frac{N_{\mathrm{std}}}{N_x} \times \frac{M_x}{M_{\mathrm{std}}} \times \frac{W_{\mathrm{std}}}{W} \times P_{\mathrm{std}}$$

I = Integral Area
N = Number of Nuclei
M = Molar Mass
W = Gravimetric Weight
P = Purity of the Analyte
x,y indicates sample and std indicates standard.

The percent of TnBPSe converted to H_2Se after 15 minutes at 250˚C was calculated from three replicate experiments.

Trial 1: 36.9%
Trial 2: 35.8%
Trial 3: 34.9%

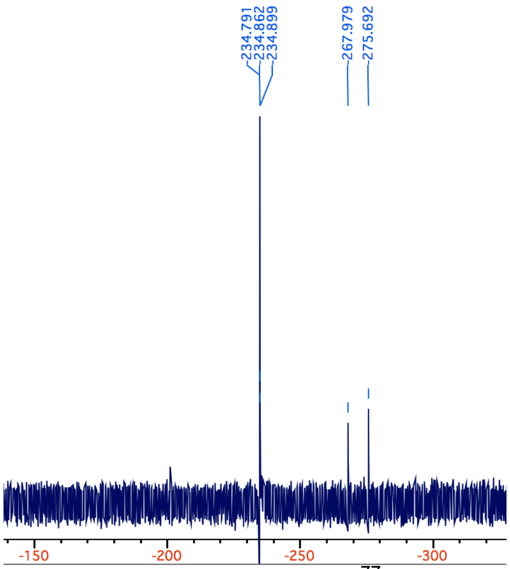

Supplementary Figure 5. ^{77}Se NMR (Right) of H_2Se collected in CS_2 through the ZnSe control experiment with a TPPSe external calibration standard. Chemical shifts are reported in Table S1.

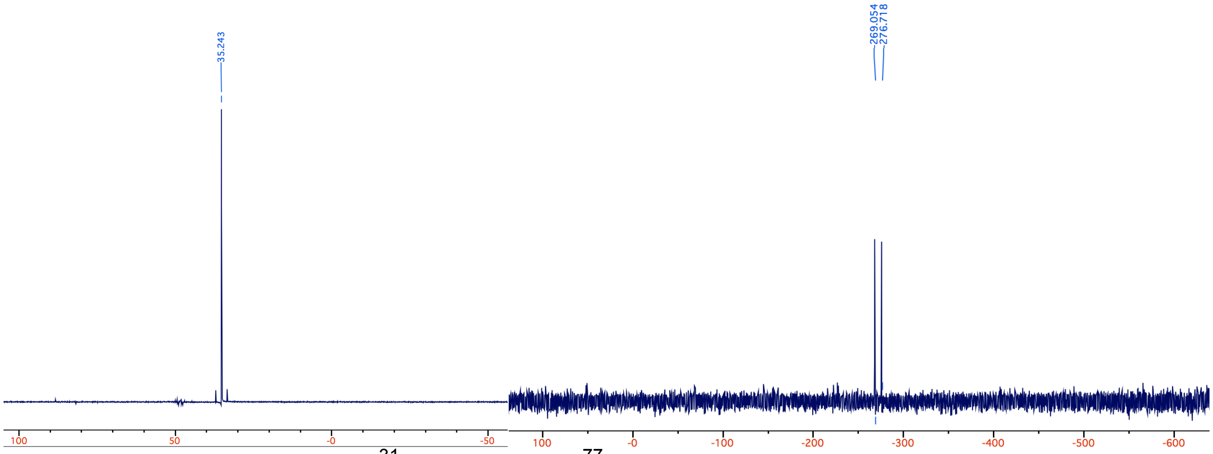

Supplementary Figure 6. ^{31}P (Left) and ^{77}Se NMR (Right) of TPPSe in CD_2Cl_2, used an external calibration standard in the quantitative ^{77}Se NMR Experiments. Chemical shifts are reported in Table S1.

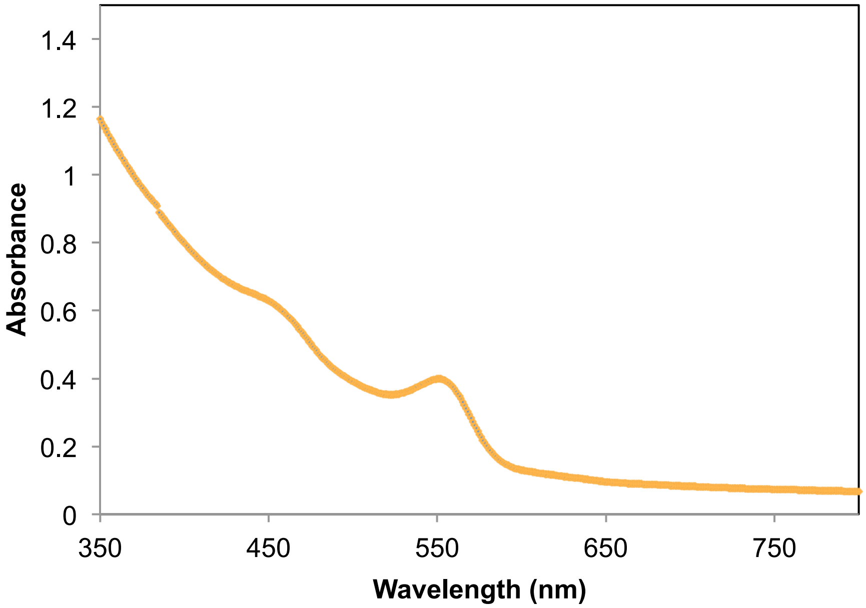

Supplementary Figure 7. Absorbance spectrum of CdSe QDs synthesized through cannula transfer of H_2Se to a flask of Cd Oleate in tetradecane at 250˚C.

Heat up method versus hot injection

Before optimizing the experiment to best mirror QD reaction conditions, a heat up method was used to observe the decomposition of TnBPSe to TnBPO. The TnBPSe, octanoic acid, and tetradecane were combined in the glove box and sealed. They were then heated to 250˚C for varying times. They were then brought back into the glovebox and transferred to NMR tubes for characterization by ^{1}H and ^{31}P NMR. This method resulted in significantly slower reaction than the hot injection approach more common to QD syntheses. Although slower, the experiment still provided relevant information regarding the decomposition of TnBPSe. Notably, in ^{1}H NMR, the acidic proton of the octanoic acid was seen to shift upfield and decrease in intensity over long reaction times, which supports our mechanism where these protons react with the phosphine selenide to form H_2Se. Additionally, the sealed reaction vials become pressurized, indicating the formation of a gas. Almost complete conversion (96%) was observed after 19 hours.

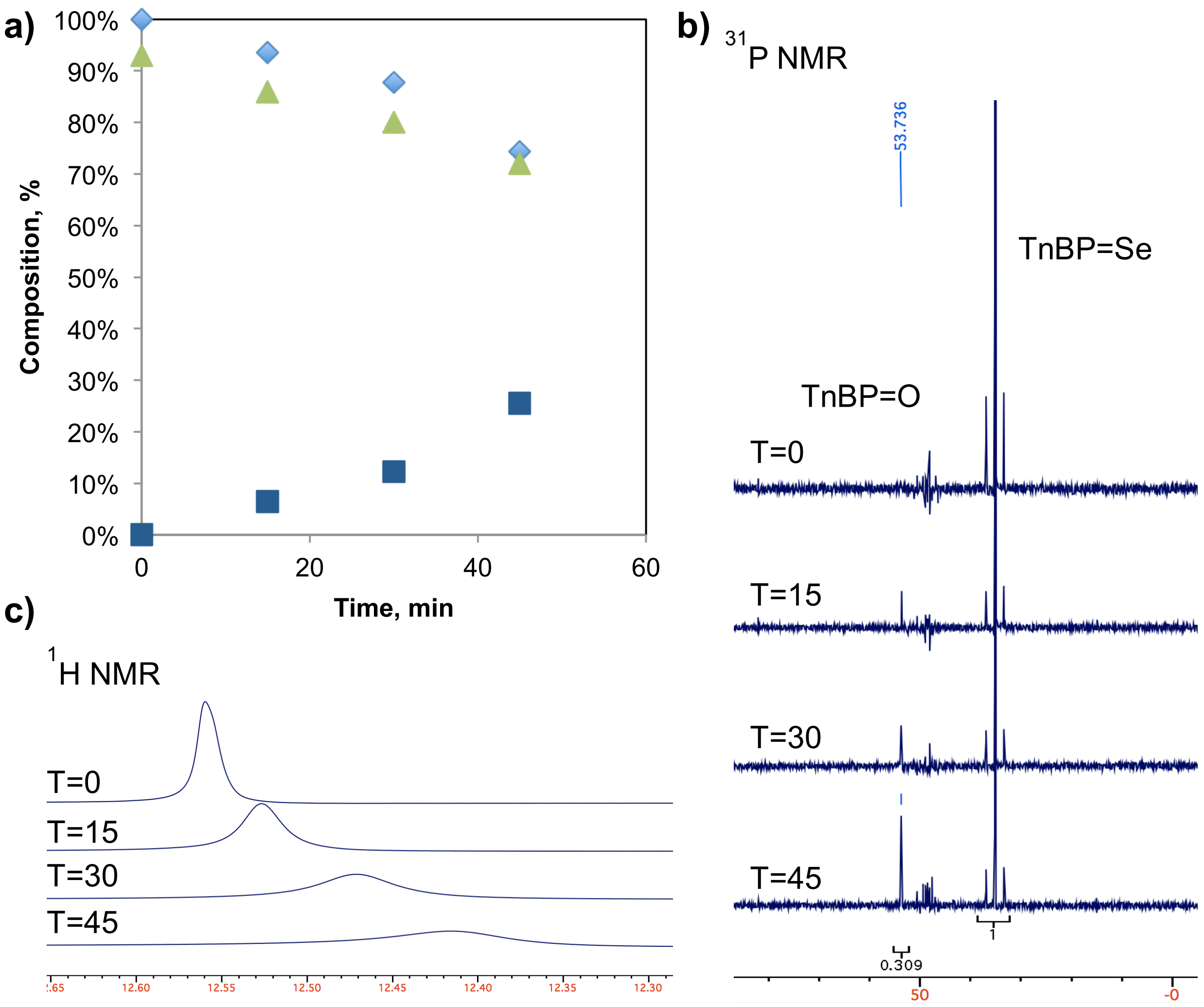

Supplementary Figure 8. TnBPSe to TnBPO conversion using heat-up method. **a)** The percent composition of TnBPSe (diamonds) and TnBPO (squares) in the TnBPSe decomposition reaction over time. The green triangles represent the loss of intensity of the acidic proton peak**.** **b)** The ^{31}P NMR spectrum of the reaction mixture over time, showing the relative amount of TnBPSe decrease as the amount of TnBPO increases. **c)** The carboxylic acid acidic proton region (12-13 ppm) in the ^{1}H NMR of the TnBPSe decomposition reaction showing the deprotonation of octanoic acid over time. Chemical shifts are reported in Table S1.

Dependence on Carboxylic Acid

The dependence of the TnBPSe decomposition on the relative concentration of octanoic acid in the reaction flask was studied by varying the excess carboxylic acid in sealed flasks as mentioned above and measuring the ^{31}P NMR after 15 minutes of reaction at 250˚C after hot injection of the TnBPSe. The results show that increased octanoic acid results in increased conversion of TnBPSe to TnBPO. Variations in the conversion percentage were seen between trials but the general trend persists.

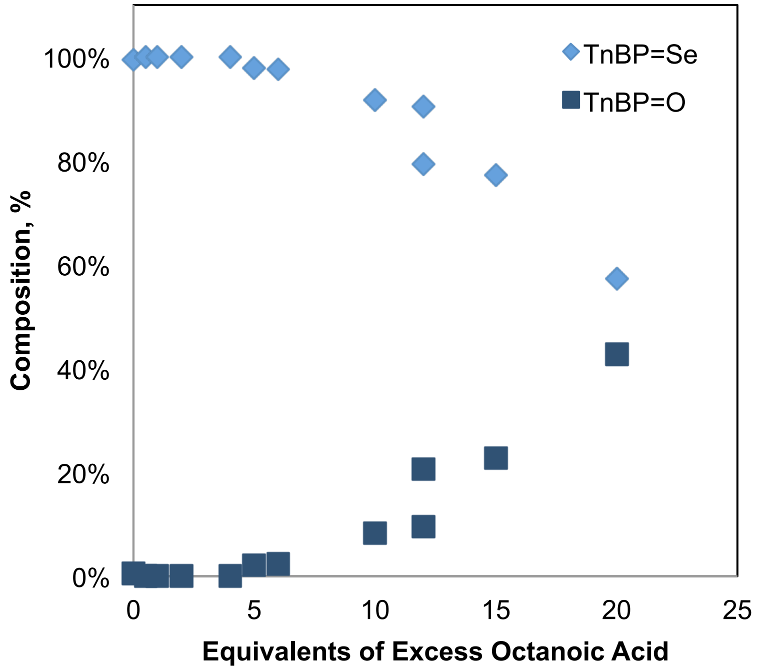

Supplementary Figure 9. The conversion of TnBPSe to TnBPO in the presence of varied amounts of excess octanoic acid after 15 minutes of reaction at 250˚C. Excess octanoic acid is any amount greater than the two equivalents necessary to make Cd octanoate.

Reaction Mechanism

The evidence provided here and in the main text lead to our proposal of the reaction mechanism below. The mechanism accounts for all known products of this reaction reported here and in the literature. The high temperatures and complicated reaction matrix of common CdSe QD syntheses were considered while devising this mechanism.

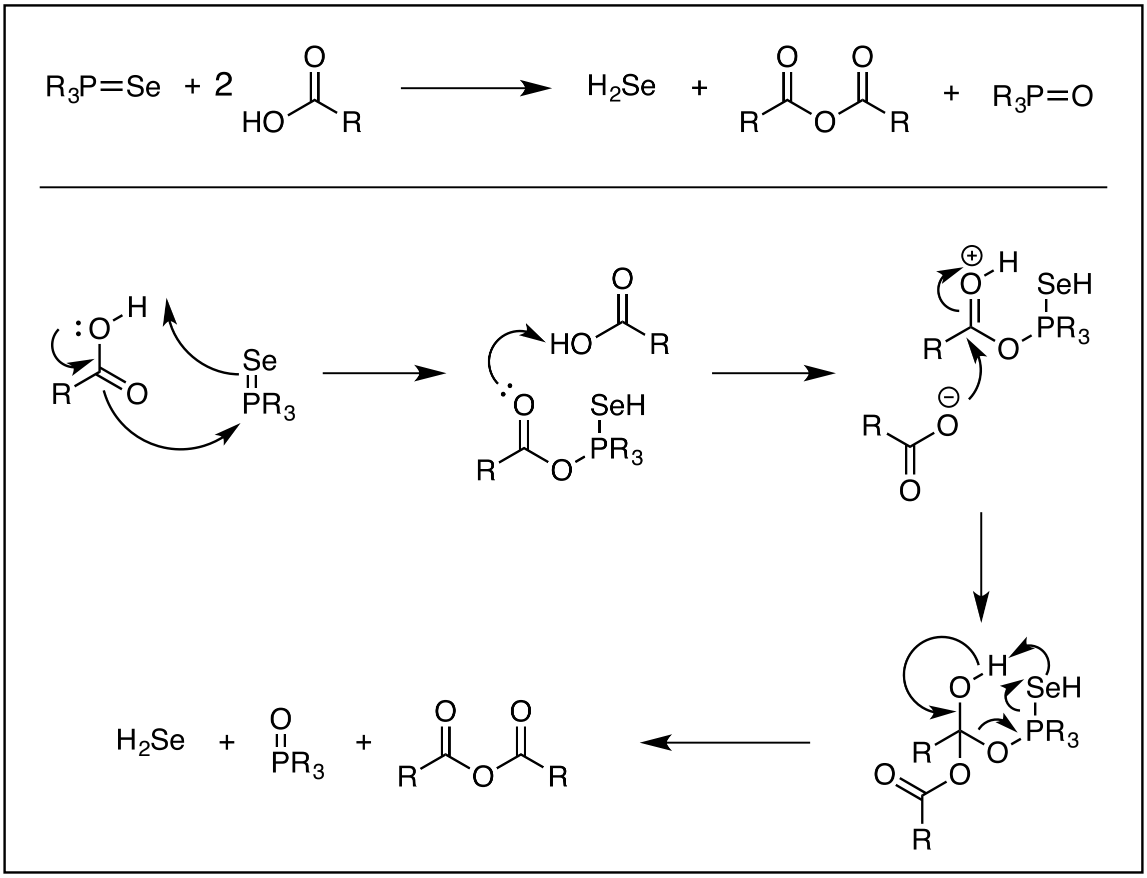

Supplementary Figure 10. Proposed reaction mechanism for H_2Se generation involving the reaction of tertiary phosphine selenides with carboxylic acid at high temperatures to yield hydrogen selenide

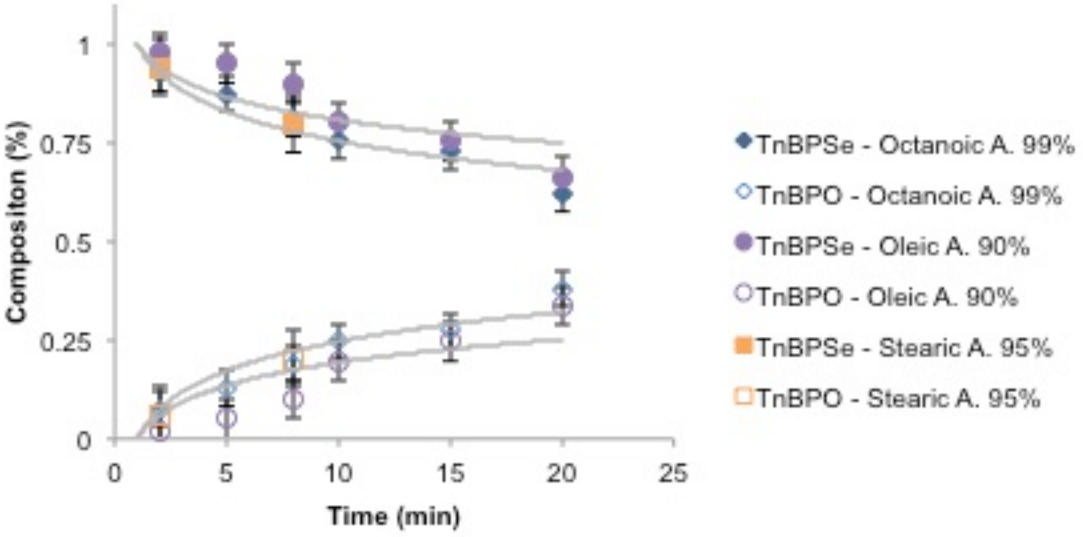

Supplementary Figure 11. A NMR analysis following decomposition of TnBPSe after hot injection. The percent composition of TXPSe (top) and TXPO (bottom) in the decomposition reaction over time in the presence of excess Oleic, Stearic, or Octanoic Acid. Trend lines are provided as guides to the eye. Error bars represent the standard error of the mean of three replicate experiments.

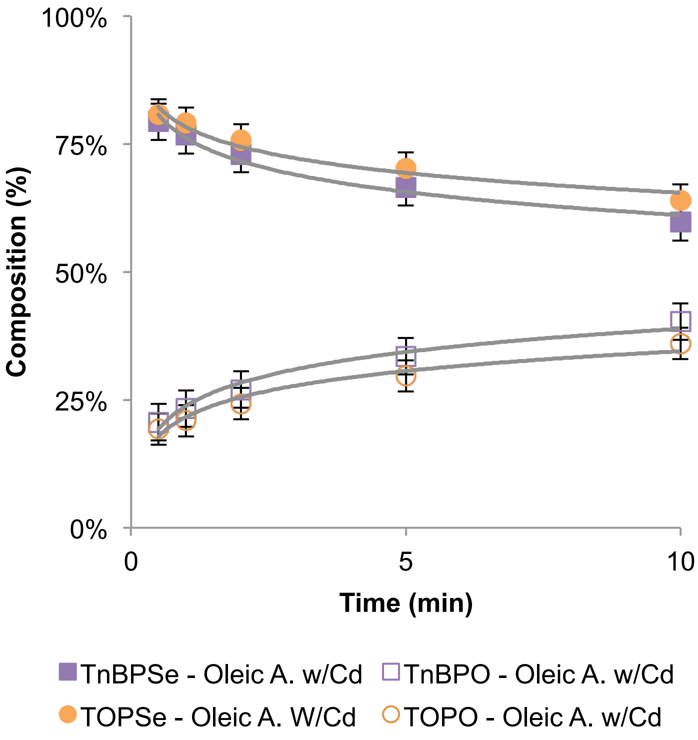

Supplementary Figure 12. A NMR analysis following decomposition of TnBPSe after hot injection. a.) The percent composition of TXPSe (top) and TXPO (bottom) in the CdSe QD forming reaction over time, comparing the reactivity of TOPSe and TnBPSe. Trend lines are provided as guides to the eye. Error bars represent the standard error of the mean of three replicate experiments.

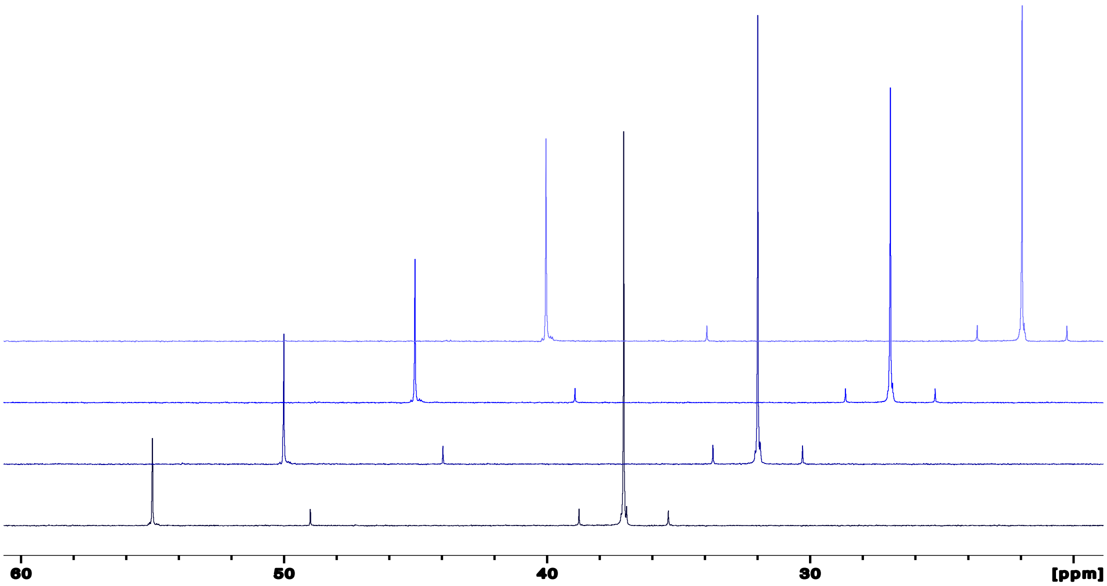

Supplementary Figure 13. ^{31}P NMR spectrum of the TOPSe reaction mixture over time, showing the amount of TOPSe (37 ppm) decreases as the amount of TOPO (55 ppm) increases. Also shown is an impurity peak at 49 ppm that does not change integration over time and is approximately 3% of the TOPSe solution. This peak is not observed with higher purity phosphines.

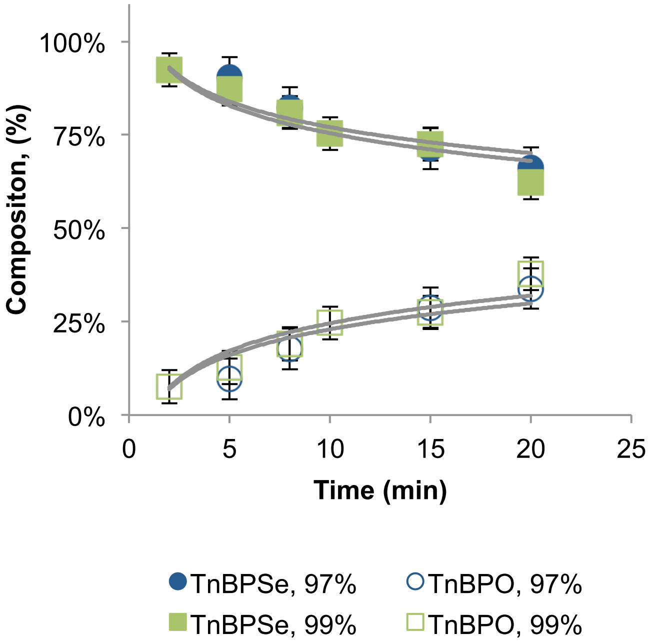

Supplementary Figure 14. A NMR analysis following decomposition of TnBPSe after hot injection. a.) The percent composition of TXPSe (top) and TXPO (bottom) in the decomposition reaction over time, comparing the reactivity of 97% and 99% TnBPSe. Trend lines are provided as guides to the eye. Error bars represent the standard error of the mean of three replicate experiments.

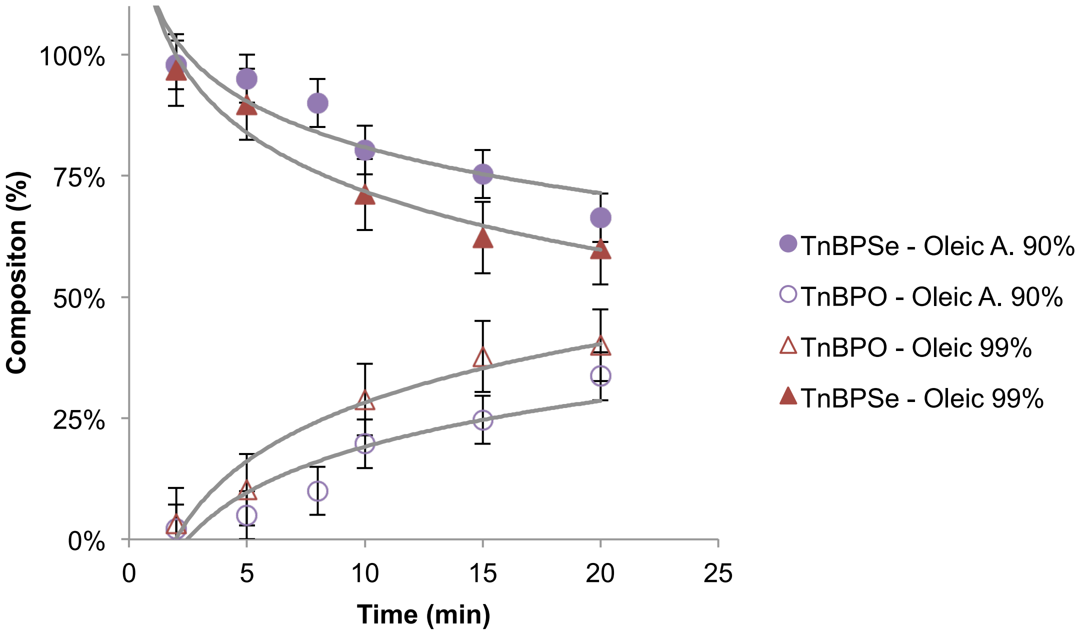

Supplementary Figure 15. A NMR analysis following decomposition of TnBPSe after hot injection. a.) The percent composition of TXPSe (top) and TXPO (bottom) in the decomposition reaction over time, comparing the reactivity of 90% and 99% Oleic Acid. Trend lines are provided as guides to the eye. Error bars represent the standard error of the mean of three replicate experiments.

Thermal decomposition of cadmium octanoate

Carboxylic acid and anhydride exchange
When different carboxylates are used in the reaction – i.e. excess oleic acid and Cd acetate, we observe a mixed anhydride species through GCMS (Supplementary Figure 16. Mixed anhydrides that are formed through the reaction of acetic anhydride and oleic acid have a higher reactivity compared to symmetric anhydrides,[5] and are in a fast equilibrium with symmetric anhydrides and free carboxylic acid (reaching equilibrium in less than 10 minutes at 100˚C).[6] This observation provides evidence that these mixed anhydrides and free carboxylic acids are available to react, perpetuating our proposed mechanism.

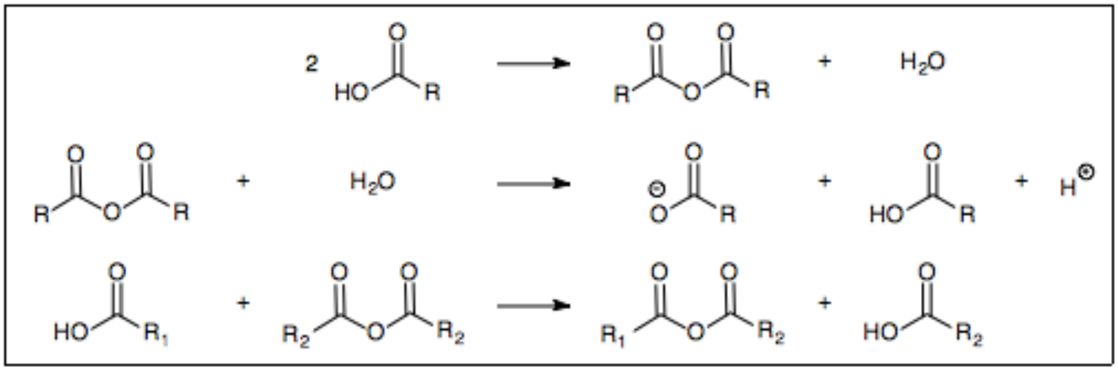

Supplementary Figure 16. Mixed anhydride exchange equilibrium

Water has been shown to increase the speed of QD formation and here it hydrolyses anhydrides into protonated and deprotonated carboxylic acids. This could contribute to the role of water in QD reactions, as it could dictate the relative concentrations of anhydride and carboxylic acid in solution. This will be a target of future studies.

^{13}C Labelled GCMS Experiment

An experiment was devised to test the hypothesis that Cd Carboxylate was also decomposing to the highly reactive alkyl cadmium. This decomposition would occur through decarboxylation of Cd carboxylate to form alkyl cadmium and carbon dioxide. Alkyl cadmium is extremely reactive, so to avoid attempting to isolate it we decided to look for the formation of carbon dioxide through a labelling study. Labelled 1-^{13}C-Octanoic acid was purchased and used to make labelled Cd octanoate, Supplementary Figure 17, line 1, which was subsequently reacted at 250˚C for 15 minutes and the headspace of the reaction was analysed by GCMS.

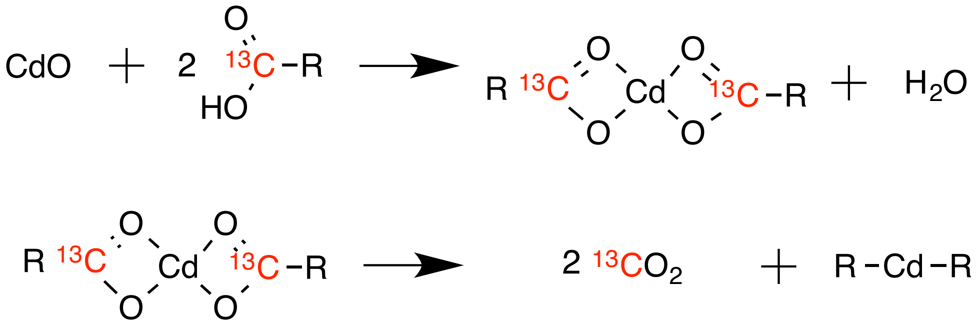

Supplementary Figure 17. Reaction scheme for ^{13}C-labeled Cd Octanoate decomposition experiment. R=C_8H_{17}

A labelled diagram of the reaction vessel can be seen in Supplementary Figure 18. The purged space in the arm of the flask was analysed to ascertain a background concentration of gasses. The Teflon pin of the flask was then opened allowing the headspace of the reaction to mix with the atmosphere in the arm of the flask. A second sample of gas was taken through the septum and the relative concentrations of gasses before and after mixing was determined.

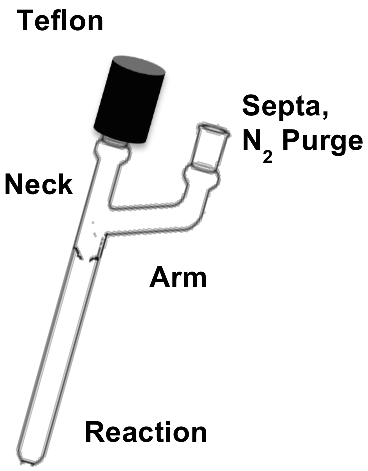

Supplementary Figure 18. Description of reaction vessel for Cd Octanoate decomposition reaction. This figure was generated using templates in ChemDraw Professional 16.0.

Alternative decomposition pathways
Another possible cadmium carboxylate decomposition at high temperatures (200-280°C) has been discussed by in the literature and also produces CO_2 as a decomposition product.[7] The proposed mechanism seen in Supplementary Figure 19 is based on the decomposition reaction of cadmium acetate in acetone where cadmium carbonate is formed and, upon further heating, evaporation of the solvent, and the release of $^{13}CO_2$, cadmium oxide is recovered.[7] This mechanism has not been studied under temperatures typical of QD synthesis conditions and has only been studied for short chain carboxylates in low boiling point solvents. The original work describes that evidence for this decomposition reaction only is observed when the decomposition rate is very slow,[8] which would not be the case under QD reaction conditions. Further, we found no evidence of cadmium carbonate or cadmium oxide forming even after 16 hours of reaction. Cadmium carbonate (white solid, insoluble in tetradecane at high temperatures) and cadmium oxide (brown precipitate) should be easily observed in the decomposition reactions in this work if they were present. Though both cadmium carbonate and cadmium oxide have been used as QD precursors, both require excess carboxylic acid or phosphonic acid to produce QDs in solution or else both are unreactive,[9] indicating that the active cadmium precursor is neither of those species.

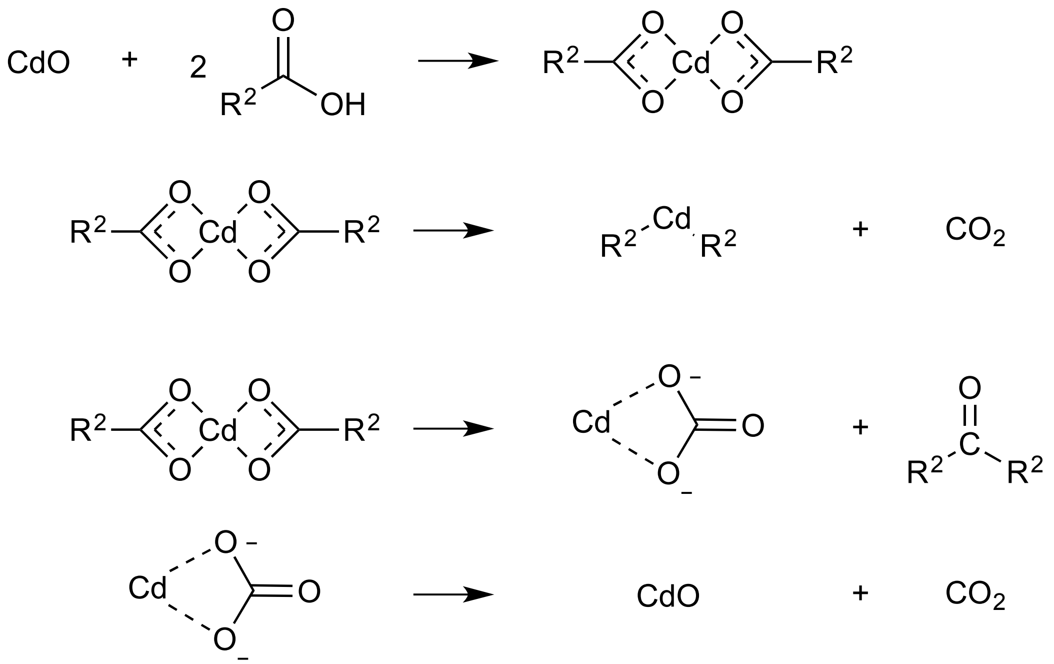

Supplementary Figure 19. Reaction scheme for alternative decomposition pathways. Line 1 & Line 2, The decomposition mechanism as we proposed. Line 3 & Line 4, The decomposition reaction proposed by Garcia-Rodriguez *et al*.[7] A ketone as shown in Line 3 is never observed in the decomposition reaction, nor are CdO and $Cd(CO)_3$, insoluble in polar solvents.

The headspace of the reaction was analysed using GCMS, and the purged space in the arm of the flask was analysed to ascertain a background concentration of gasses. The Teflon pin of the flask was then opened allowing the headspace of the reaction to mix with the atmosphere in the arm of the flask. A second sample of gas was taken through the septum and the relative concentrations of gasses before and after mixing was determined. Proper technique is critical to ensure minimal contamination from atmospheric CO_2. ^{13}C-labeled cadmium carboxylate was synthesized from 1-^{13}C-octanoic acid and cadmium oxide in tetradecane by heating to 210°C for 30 minutes in an schlenk flask (Supplementary Figure 19. The resulting clear colourless liquid was degassed and the arm of the flask was sealed with a septum and purged with nitrogen. The sealed flask was then heated to 280°C for 15 minutes.

A calibration curve was created by varying the amounts of $^{13}CO_2$ added to a schlenk flask filled with an equivalent volume of tetradecane as in the experimental unknowns with an argon internal standard that was added equally across all samples. The ratio of counts versus the internal standard was calculated by dividing the counts of $^{13}CO_2$ (m/z=45) by those of Ar (m/z = 40). The average for each concentration was taken over five samples.

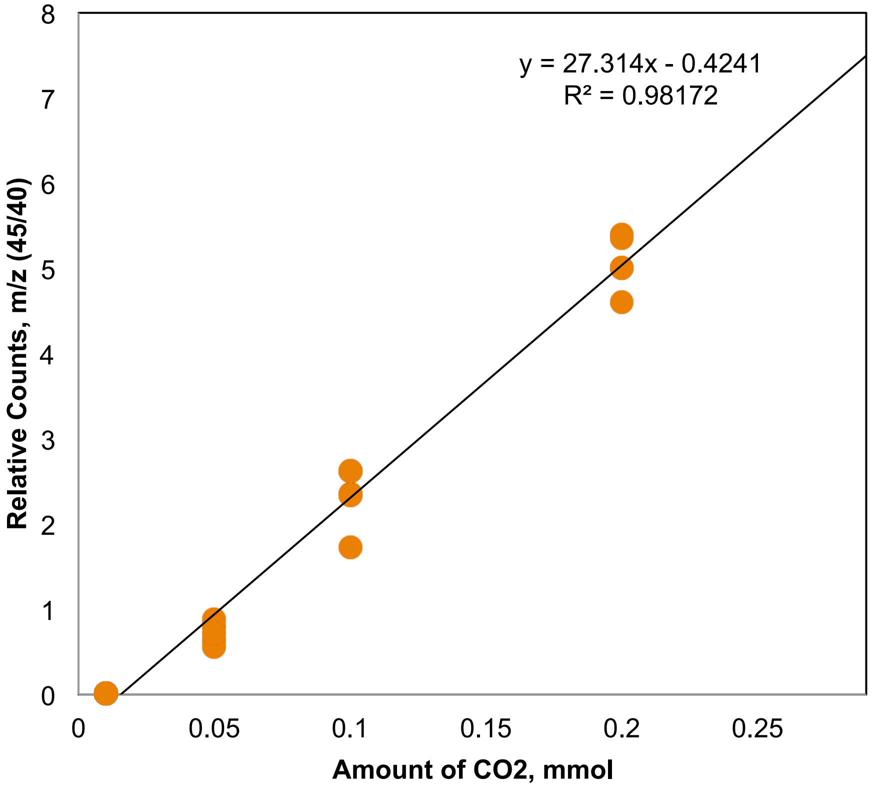

Supplementary Figure 20. GCMS Calibration curve of $^{13}CO_2$ normalized to an argon external standard. Five replicates of each sample were taken and the linear fit was used to calculate the amount of $^{13}CO_2$ detected.

Supplementary Table 2. Amount of $^{13}CO_2$ Detected by GCMS

Experiment	CO_2 (mmol)	Percent Conversion
QD Conditions 15 min	0.029	5%
QD Conditions 16 hours	0.032	6%
QD Synthesis 15 min	0.029	5%
Stoichiometric 15 min	0.297	48%

QD conditions include 12 times excess 1-^{13}C-Octanoic acid, standard to a QD synthesis where excess carboxylic acid is used as a ligand on the QD surface as well as to solubilize reactants. The QD synthesis included the addition of TnBPSe at the start of the reaction and QDs were formed through the heat up method in the same reaction flask (Supplementary Figure 18). The absorbance spectrum of the resulting QDs is depicted in Supplementary Figure 21.

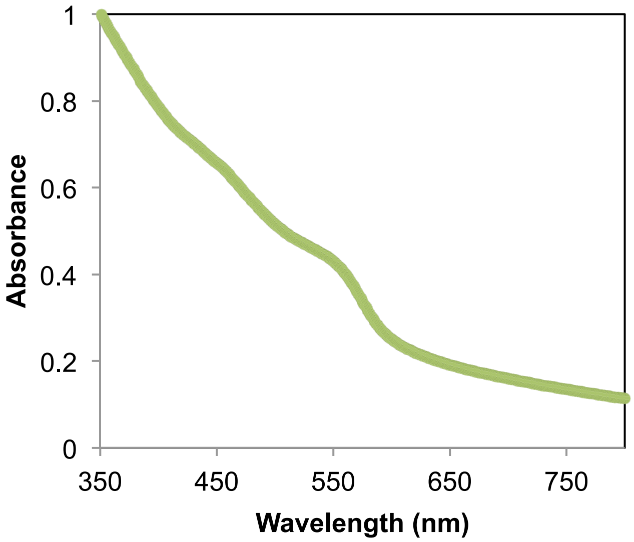

Supplementary Figure 21. Absorbance spectrum of CdSe QDs synthesized in the reaction vessel (Supplementary Figure 18) through the heat-up method at 250˚C. The headspace of this reaction was analysed by GCMS as reported in Figure 5 of the main text.

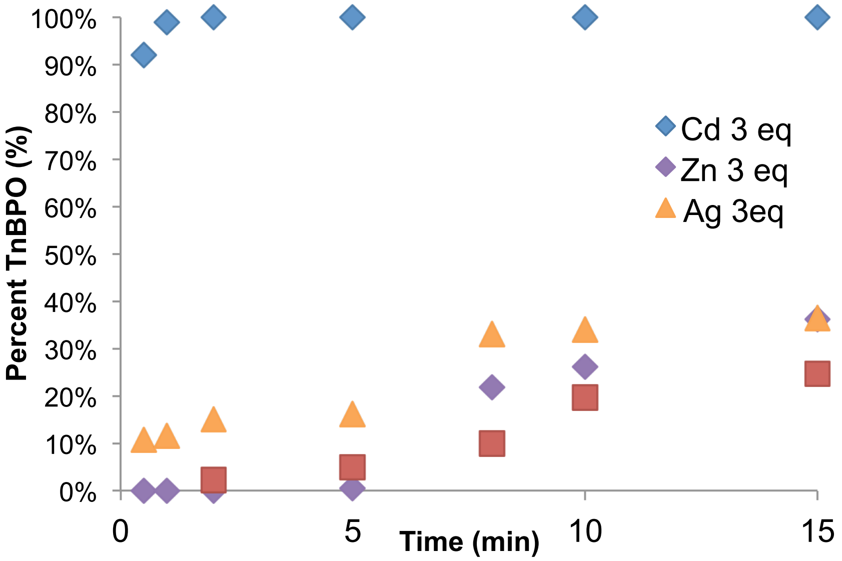

Supplementary Figure 22. A NMR analysis following conversion of TnBPSe to TnBPO after hot injection. The percent composition TXPO in the decomposition reaction over time is shown, comparing the reactivity with three equivalents of cadmium, silver, or zinc oleate to that of the reaction in the absence of metal.

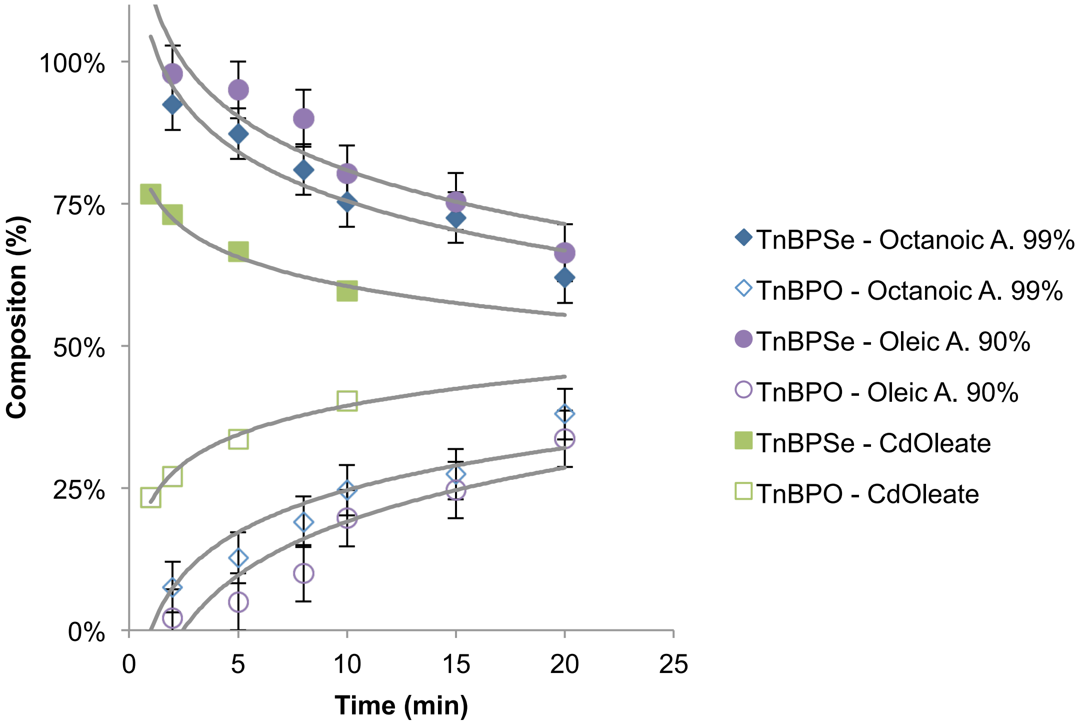

Supplementary Figure 23. A NMR analysis following decomposition of TnBPSe after hot injection. The percent composition of TXPSe (top) and TXPO (bottom) in the decomposition reaction over time, comparing the reactivity with and without cadmium present. Trend lines are provided as guides to the eye. Error bars represent the standard error of the mean of three replicate experiments.

The low $^{13}CO_2$ conversion efficiencies could be augmented by considering the percentage of labelling of the 1-^{13}C-octanoic acid starting material. A ^{13}C NMR of the 1-^{13}C-octanoic acid as purchased showed a 62% ^{13}C labelling of the carboxylic acid carbon. This would nearly double the percent conversion and could be an experimental source of error for these calculations.

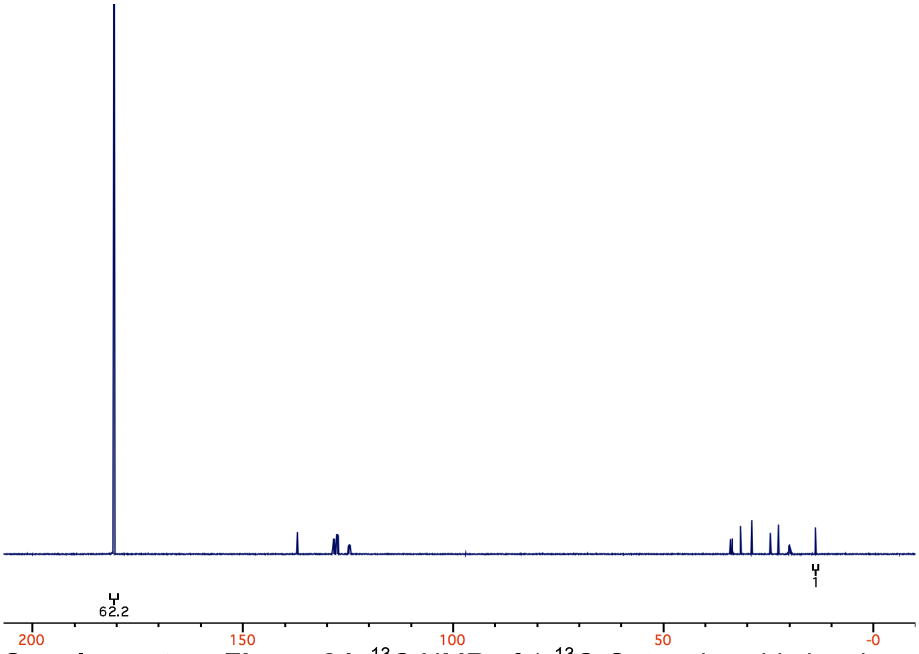

Supplementary Figure 24. ^{13}C NMR of 1-^{13}C-Octanoic acid showing only 62% ^{13}C labelling.

Supplementary References

1. Schneider, R., Weighardt, K. & Nuber, B. New p-Disulfido and p-Diselenido Complexes of Ruthenium(111). Crystal Structure of [(LRum(acac))2(p-S2)]P(F& (L = 1,4,7-Trimethyl-1,4,7-triazacyclononane;acac = Pentane-2,4-dionate). *Inorg. Chem.* **32,** 4935–4939
2. Birchall, T., Gillespie, R. J. & Verkris, S. L. Nuclear Magnetic Resonance Spectroscopy of Some Selenium Compounds. *Can. J. Chem.* **43,** 1672–1679 (1965).
3. Alvarado, S. R., Shortt, I. A., Fan, H.-J. & Vela, J. Assessing Phosphine–Chalcogen Bond Energetics from Calculations. *Organometallics* **34,** 4023–4031 (2015).
4. Bharti, S. K. & Roy, R. Quantitative. *Trends in Analytical Chemistry* **35,** 5–26 (2012).
5. Edwards, W. R. & Sibille, E. C. Mixed Carboxylic Anhydrides in the Friedel-Crafts Reaction. *J. Org. Chem.* **28,** 674–679 (1963).
6. Peydecastaing, J., Vaca-Garcia, C. & Borredon, E. Consecutive Reactions in an Oleic Acid and Acetic Anhydride Reaction Medium. *Eur. J. Lipid Sci. Technol.* **111,** 723–729 (2009).
7. García-Rodríguez, R., Hendricks, M. P., Cossairt, B. M., Liu, H. & Owen, J. S. Conversion Reactions of Cadmium Chalcogenide Nanocrystal Precursors. *Chem. Mater.* **25,** 1233–1249 (2013).
8. Akanni, M. S., Okoh, E. K., Burrows, H. D. & Ellis, H. A. The thermal behaviour of divalent and higher valent metal soaps: a review. *Thermochimica Acta* **208,** 1–41
9. Qu, L., Peng, Z. A. & Peng, X. Alternative Routes toward High Quality CdSe Nanocrystals. *Nano Lett.* **1,** 333–337 (2001).
